# OpenCap vs. LESS: Sport-Specific Profiling of Dynamic Knee Valgus

**DOI:** 10.3390/jcm14248879

**Published:** 2025-12-15

**Authors:** Ji-Hoon Cho, Seung-Taek Lim, An-Sik Heo

**Affiliations:** 1 Department of Sports Medicine, Dongshin University, Naju 58245, Republic of Korea; jhchopro@naver.com; 2 College of General Education, Kookmin University, Seoul 02707, Republic of Korea; 3 Waseda Institute for Sport Sciences, Waseda University, Saitama 341-0018, Japan

**Keywords:** Landing Error Scoring System, dynamic knee valgus, markerless 3D, EMG, anterior cruciate ligament, biomechanics

## Abstract

**Background/Objectives**: This study investigated the relationships among the Landing Error Scoring System (LESS), markerless 3D (OpenCap)-derived knee valgus, and surface electromyography (EMG) of quadriceps and hamstrings. **Methods**: Thirty-two healthy male university students (26 athletes, 6 non-athletes) completed a drop-landing task. LESS was video-scored; knee valgus at peak knee flexion was computed with OpenCap; and bilateral rectus femoris (RF) and biceps femoris (BF) EMG was recorded. Phase-specific EMG was normalized to peak dynamic activity. **Results**: LESS showed an independent negative association with left knee valgus (*p* = 0.001). In the regression model, bilateral BF acceleration-phase activity was a significant predictor of knee valgus (*p* < 0.05). Exploratory comparisons indicated that athletes exhibited lower RF deceleration activity and smaller left-side valgus than non-athletes. **Conclusions**: These findings suggest that hamstring activity during the transition phase is linked to knee alignment. This study demonstrates the complementary value of integrating LESS with markerless 3D motion capture, although caution is warranted when generalizing group differences due to the unequal sample size.

## 1. Introduction

Non-contact knee injuries—particularly anterior cruciate ligament (ACL) injuries—remain a major concern in sport due to delayed return to play, reduced performance, and long-term consequences for joint health among young athletes. Dynamic knee valgus (DKV) has been widely identified as a key at-risk movement pattern, with recent reviews emphasizing that valgus alignment during landing and change-of-direction tasks, together with horizontal deceleration demands, is closely associated with ACL loading (anterior tibial shear; combined valgus–rotation moments) [1,2].

The Landing Error Scoring System (LESS) is a widely used field screening tool that captures landing errors—including medial knee collapse, trunk sagittal/frontal deviations, and foot strike patterns—in a practical manner. Systematic reviews indicate generally good reliability and construct validity for LESS, with increasing evidence of convergence with 3D motion analysis [3]. Emerging work linking trunk and hip function to LESS underscores that it reflects whole-body coordination quality rather than being solely a “knee-only” metric [4]. Collectively, these findings motivate studies that directly probe the link between LESS and 3D alignment indices (e.g., knee valgus angle/moment).

Simultaneously, markerless 3D motion capture has improved rapidly in validity and reliability, enabling study designs that integrate field screening (LESS) with quantitative 3D metrics from the same movement. Platforms such as OpenCap leverage multi-camera, deep-learning-based pose estimation to estimate lower-extremity kinematics with clinically useful accuracy in field and clinical settings [5,6]. Reviews addressing implementation highlight its potential as a practical “bridge” between screening, training, and rehabilitation, provided that camera setup, algorithm choice, and validation pipelines are standardized [7].

Landing performance is also influenced by limb-side strategies, sport-specific demands, and external task constraints (e.g., footwear, loading, surface), each of which can modulate knee responses. Experimental studies demonstrate that attentional focus and load conditions influence knee valgus/varus responses and ground-reaction profiles [8], while reviews emphasize the central role of hamstrings and posterior-chain coordination in horizontal deceleration and joint stabilization [1,9]. Side-to-side differences in landing mechanics have likewise been reported, suggesting that limb dominance and support roles may modulate valgus-related loads [10]. Against this background, the primary aim of this study was to examine the coupling among LESS scores, 3D valgus alignment, and EMG timing within a single integrated framework. Specifically, we addressed two research questions: (1) Does the field-based LESS score correlate with quantitative 3D knee valgus measured by a markerless system and (2) How do neuromuscular strategies (EMG) relate to these kinematic patterns? Additionally, we conducted an exploratory analysis to observe potential differences in landing strategies between trained athletes and non-athletes.

## 2. Methods

### 2.1. Participation

This observational cross-sectional study included 32 healthy male university students in their 20s (athletes: *n* = 26, comprising 18 soccer and 8 taekwondo athletes; non-athletes: *n* = 6). Male participants were selected to control for sex-related neuromuscular differences (e.g., Q-angle, hormonal fluctuations) in this preliminary investigation. All participants provided written informed consent after receiving a full explanation of the study aims, procedures, and potential risks and benefits. Inclusion criteria were no lower-limb injury within the past 6 months and willingness to complete all procedures. Exclusion criteria were non-correctable orthopedic conditions (e.g., congenital deformities), diagnosed sleep disorders, and neurological or cardiovascular contraindications. Stature, body mass, and self-reported limb dominance were recorded. A standardized warm-up (5 min cycling plus dynamic stretching) preceded testing. Six non-athletes were included a priori to provide a reference range. We acknowledge the unequal sample size (*n* = 26 vs. *n* = 6); therefore, all comparisons between athletes and non-athletes are interpreted as exploratory and descriptive rather than confirmatory.

### 2.2. Landing Error Scoring System (LESS)

LESS was evaluated using a drop-landing task from a 30 cm box followed by an immediate vertical rebound jump. Two cameras (frontal and sagittal views) recorded the task. Two experienced raters, who were blinded to the participants’ group allocation, scored independently; the mean of their scores was used, with higher scores indicating more landing errors [1]. Before data collection, raters calibrated their criteria using the Padua et al. training video and jointly scored 10 pilot trials to align decision rules [11].

### 2.3. Markerless 3D Motion Analysis (OpenCap) and Knee Valgus

Markerless 3D kinematics were estimated with OpenCap (https://www.opencap.ai/ accessed on 4 November 2025). Video data were captured using two smartphone cameras (iOS, 1080p, 60 fps) placed 3.5 m from the participant at a 45-degree angle relative to the frontal plane at a lens height of ~1.0 m [6]. A static T-pose was used for calibration. Although markerless systems may exhibit higher variability in the coronal plane compared to marker-based systems, OpenCap has demonstrated moderate-to-good validity (r > 0.9 for flexion, RMSE < 5 degrees) in dynamic tasks [6]. To minimize noise, joint angle data were processed using a 4th-order low-pass Butterworth filter (cutoff frequency 6 Hz) inherent to the OpenCap pipeline. Knee valgus angle (coronal alignment) was defined at peak knee flexion after initial contact; according to our sign convention, more negative values indicate greater valgus.

### 2.4. Surface Electromyography (EMG)

Surface EMG was recorded bilaterally from the rectus femoris (RF) and biceps femoris long head (BF) following SENIAM/CEDE guidelines (shaving, abrasion, alcohol prep; electrode pairs 2 cm apart, aligned with fiber direction) [12]. Wireless EMG was sampled at 1000 Hz, band-pass-filtered (20–450 Hz), notch-filtered (50/60 Hz as needed), full-wave-rectified, and low-pass-filtered at 6 Hz (4th-order zero-lag Butterworth) to obtain linear envelopes. EMG signals were normalized to the peak root mean square (RMS) value obtained during the landing task itself (dynamic normalization), as maximum voluntary contraction (MVC) tests were not feasible in this field-based protocol. This method allows for the assessment of relative muscle contribution during the specific movement.

### 2.5. Phase Definitions (Deceleration/Acceleration)

The interval from initial contact to peak knee flexion was defined as deceleration, and from peak flexion to take-off as acceleration [1]. In the absence of force plates, initial contact (IC) and toe-off (TO) events were identified using a kinematic algorithm based on the vertical velocity of the heel marker and visual verification. Specifically, IC was defined as the frame where the heel vertical velocity dropped below a threshold of 0.1 m/s, confirmed by frame-by-frame visual inspection of foot deformation on the video. For each phase, RMS EMG (50 ms window) was computed. Phase-specific RF/BF ratios (acceleration, deceleration) were derived to index relative quadriceps–hamstring contributions.

### 2.6. Statistical Analysis

Analyses were conducted in SPSS 25.0 (IBM, Armonk, NY, USA). Data are reported as mean ± SD. Group differences (soccer, taekwondo, non-athletes) were tested with one-way ANOVA and Bonferroni-corrected post hoc comparisons. Pearson correlations tested bivariate associations. Multiple linear regression modeled: (1) LESS as the dependent variable with left/right valgus as predictors; (2) valgus (right side) as the dependent variable with bilateral RF/BF acceleration (Acc) and deceleration (Dec) indices as simultaneous predictors. Multicollinearity was assessed using Variance Inflation Factors (VIF < 5.0). We report unstandardized coefficients (B, 95% CI), standardized β, SE, t, p, and model R^2^/adjusted R^2^. Associations between LESS and valgus were summarized with Pearson’s r and 95% CIs (Fisher’s z). Side-to-side asymmetry was quantified by the paired mean difference (L–R) with 95% CIs and Cohen’s d (mean difference divided by the SD of paired differences). Athlete vs. non-athlete summaries were descriptive/exploratory. Significance was set at two-tailed α = 0.05. Given the total sample size (*N* = 32) and the imbalance in group sizes (non-athletes, *n* = 6), a post hoc sensitivity analysis indicated that the study was powered to detect only large effects for between-group comparisons (e.g., capable of detecting correlations of r ≥ 0.48 with 80% power). Additionally, for the multiple regression models, the number of predictors was relatively high compared to the sample size; thus, regression coefficients should be interpreted with caution. Therefore, all comparisons involving the non-athlete group should be interpreted as exploratory and hypothesis-generating, acknowledging the risk of Type II error due to limited statistical power.

## 3. Results

### 3.1. Group Comparisons

**Figure 1:**
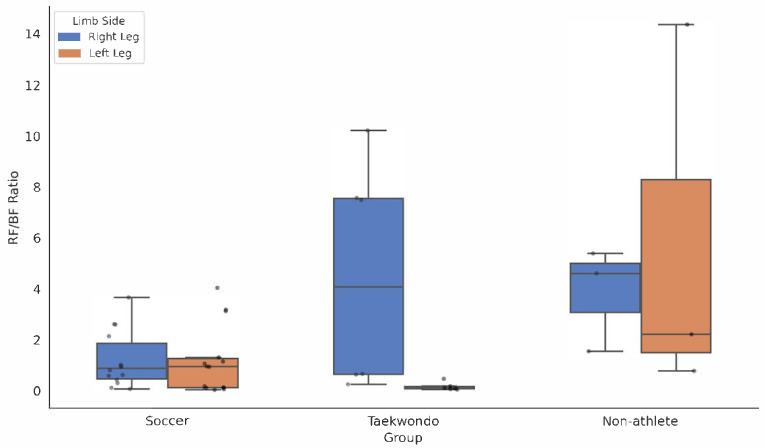
Comparison of the Rectus Femoris to Biceps Femoris (RF/BF) ratio during the deceleration phase across groups. The boxplots illustrate the median and interquartile ranges, while individual data points represent subject variability. Note the significantly higher ratios and variability in the Non-athlete group compared to the athlete groups, indicating a quadriceps-dominant landing strategy.

To supplement the *p*-values, effect sizes (partial eta-squared, η^2^) were calculated for all ANOVA comparisons to determine the practical significance of the observed differences (Table 1). Across soccer, taekwondo, and non-athlete groups, LESS scores did not differ significantly (η^2^ = 0.056). On the right side, there were no group differences in knee valgus angle, RF/BF acceleration-phase EMG, or the RF/BF acceleration ratio. However, soccer athletes showed lower RF deceleration EMG than taekwondo athletes (*p* < 0.05), demonstrating a large effect size (η^2^ = 0.239), and a lower RF/BF deceleration ratio than non-athletes (*p* < 0.05, η^2^ = 0.226). On the left side, soccer athletes exhibited a smaller (i.e., more stable) valgus angle than non-athletes (*p* < 0.05), representing a large effect (η^2^ = 0.153); and both RF deceleration EMG and the RF/BF ratios (acceleration and deceleration) were lower in soccer and taekwondo athletes than in non-athletes (*p* < 0.05), showing large effect sizes (η^2^ ranging from 0.204 to 0.285). Preliminary exploratory comparisons suggested that soccer and taekwondo athletes may exhibit lower RF deceleration activity, lower RF/BF ratios, and smaller left-side valgus than non-athletes. However, these findings should be viewed with caution given the small non-athlete sample (*n* = 6) and are consistent with a potentially more stable landing strategy (Figure 1).

### 3.2. Correlations with LESS

By our sign convention (more negative values indicate greater valgus), LESS correlated negatively with right-side valgus (r = −0.453, *p* = 0.018) and with the right-side RF/BF acceleration ratio (r = −0.541, *p* = 0.008) (Table 2). Other right-side EMG indices were not significant. On the left side, LESS showed a strong negative correlation with valgus (r = −0.662, *p* < 0.001), whilst correlations with EMG variables and RF/BF ratios were not significant. Thus, higher LESS scores were associated with greater (more negative) valgus, particularly on the left, and with a lower RF/BF acceleration ratio on the right.

### 3.3. Multiple Regression with LESS as the Dependent Variable

Regression of LESS on left and right valgus explained a substantial portion of variance (R^2^ = 0.491; adjusted R^2^ = 0.448) (Table 3). Left valgus was a significant independent predictor (B = −0.308, 95% CI −0.481 to −0.135; SE = 0.084; β = −0.574; t = −3.668; *p* = 0.001), whilst right valgus did not reach significance (B = −0.072, 95% CI −0.167 to 0.023; SE = 0.046; β = −0.245; t = −1.564; *p* = 0.131). Interpreting the sign convention, higher LESS scores were independently associated with greater (more negative) valgus on the left side only.

### 3.4. Multiple Regression with Right-Side Valgus as the Dependent Variable

Regression of right-side valgus on bilateral RF and BF EMG indices for Acc and Dec phases yielded a model with R^2^ = 0.650 (adjusted R^2^ = 0.387) (Table 4). BF acceleration was a significant bilateral predictor: right BF Acc (B = −0.021, 95% CI −0.041 to −0.001; SE = 0.009; β = −0.698; t = −2.237; *p* = 0.045) and left BF Acc (B = −0.020, 95% CI −0.039 to 0.000; SE = 0.009; β = −0.633; t = −2.211; *p* = 0.047). Right RF Acc, right RF Dec, right BF Dec, left RF Acc, left RF Dec, and left BF Dec were not significant (all *p* > 0.05). Because more negative values reflect greater valgus, these negative coefficients indicate that higher BF acceleration-phase activity was associated with greater (more negative) right-side valgus, while other EMG predictors did not contribute independently.

## 4. Discussion

This study identified three principal findings. First, LESS was independently associated with left knee valgus, whereas the association with the right side was not significant. Second, in the regression model for valgus, bilateral hamstring acceleration-phase activity (biceps femoris, BF Acc) emerged as a negative predictor. However, given the limited sample size relative to the number of predictors, non-significant associations in the model may reflect a lack of statistical power rather than a true absence of effect. Third, exploratory comparisons suggested that soccer and taekwondo athletes—relative to non-athlete students—tended to show lower rectus femoris (RF) deceleration activity, lower RF/BF ratios, and smaller left-side valgus. Although preliminary, these patterns may reflect a more “stable” landing pattern. Below, we interpret these patterns, underlying mechanisms, and clinical implications in the context of recent evidence.

Evidence supporting the validity of LESS as a field tool to capture at-risk movements such as dynamic knee valgus (DKV) during jump–landing has continued to accumulate. Recent scoping reviews have systematized variants (e.g., LESS-M) and sex- or sport-specific reference values, reinforcing LESS as a widely used screening instrument [13]. Although overall reliability and construct validity are favorable, some authors note room for improvement in precise agreement and predictive validity relative to 3D motion capture [3]. Modified tools (LESS-M, SL-LESS) demonstrate at least moderate convergence with 3D metrics, strengthening the emerging “bridge” between field screening and 3D quantification [14,15].

In the present study, the observation of the LESS–valgus coupling only on the left side is plausibly explained by that limb acting as the primary support/braking side during task execution, facing greater demands for alignment stabilization immediately after landing. Recent work indicates that the more heavily utilized limb can exhibit larger peak knee abduction moments and lateral ground-reaction loading [10], and that patients after ACL reconstruction display side-dependent differences in neuromuscular control and landing mechanics during drop landings [16]. Because energy absorption during landing can be distributed unequally across limbs [8], a stronger coupling between LESS and valgus on one side is defensible. Overall, the more pronounced LESS–valgus association on the left likely reflects side-specific strategy differences imposed by task demands.

The finding that bilateral BF Acc negatively predicts right-side valgus highlights the important role of hamstrings during the landing-to-reacceleration transition. Hamstrings reduce anterior tibial shear and can offset quadriceps-dominant anterior shear that elevates ACL load—a principle recognized across mechanistic and clinical literature [17]. Framed temporally, preactivation and early stiffness of the hamstrings are decisive for alignment control during the rapid deceleration–re-acceleration transition, a point repeatedly emphasized in recent syntheses of horizontal deceleration demands [1]. Experimental work also shows that greater hamstring preactivation immediately before and after landing is linked to more stable early landing responses [18]. Accordingly, our observed association—higher BF Acc with greater (more negative) valgus—likely does not indicate that hamstring activity worsens valgus. Rather, valgus may occur first, followed by reactive increases in acceleration-phase hamstring activity as a compensatory response. Thus, when timing is suboptimal, surface EMG may appear elevated in the acceleration phase because correction occurs only after valgus has already developed. Notably, BF Acc appeared as a predictor bilaterally, suggesting a coordinated compensatory mechanism. High-speed synchronized analyses combining ground-reaction forces, 3D kinematics, and EMG are needed to clarify event timing and to distinguish protective preactivation from reactive compensation.

The notion that greater valgus biases energy absorption toward the knee is consistent with prior findings. In valgus-aligned landings, hip contribution (negative work) tends to decrease while the knee’s absorption burden increases [19]. In our athlete groups (soccer/taekwondo), the combination of lower RF deceleration, lower RF/BF ratios, and smaller left-side valgus suggests reduced reliance on knee-centric deceleration and a shift toward a posterior-chain-dominant strategy that redistributes absorption through the gluteal–hamstring complex. This interpretation aligns with contemporary views emphasizing coordinated contributions of hip extensors and ankle plantar flexors for horizontal deceleration and joint protection [1]. A meta-analysis has further reported compensatory increases in lower-limb muscle activation—including hamstrings—among individuals with DKV [20], supporting our interpretation that the observed BF Acc increase with greater valgus reflects a compensatory signature rather than a causal aggravation.

With respect to side-specificity, energy and load sharing during landing are not necessarily symmetrical. Depending on task, training background, and habitual movement, one limb may assume a predominant support/braking role, yielding a stronger LESS–valgus coupling on that side. Clinical data show side-dependent differences in neuromuscular control and landing mechanics after ACL reconstruction [17], and experimental manipulations (e.g., attentional focus) can modulate knee responses during landing [8]. Our findings—robust coupling between screening (LESS) and 3D alignment (valgus) on the left, alongside timing-weighted compensation (BF Acc) emphasized on the right—fit this asymmetric framework. Future multivariate models incorporating explicit limb designation and static and dynamic symmetry metrics could clarify these asymmetries and their practical significance.

Methodologically, the validity and reliability of markerless 3D systems such as OpenCap are improving rapidly, increasing their utility as a bridge between field screening (LESS) and quantitative kinematics [21,22,23]. Nevertheless, results can be sensitive to how acceleration and deceleration phases are defined (landing–peak flexion–re-acceleration boundaries), to signal processing choices, and to camera configuration or algorithmic parameters affecting joint-angle estimation. Careful standardization of timing criteria and enhanced synchronization precision are therefore warranted in studies adopting similar designs.

From a practical standpoint, combining LESS with streamlined markerless 3D kinematics is a sensible approach to flag left-dominant DKV risk early and to guide training that targets hamstring preactivation and early stiffness for deceleration–re-acceleration control [1,18,19]. The athlete profile observed here—lower RF deceleration, lower RF/BF ratio, and smaller valgus—supports posterior-chain-biased coaching for non-athletes and developing players. Finally, side-specific monitoring and complementary training—such as task assignment, unilateral loading, and fatigue management—should be incorporated into a multivariate framework that jointly tracks LESS, valgus alignment, and EMG timing indices to refine individualized risk mitigation. Several limitations must be acknowledged. First, the sample size of the non-athlete group (*n* = 6) was small compared to the athlete group. Because of this imbalance, between-group comparisons are underpowered and the estimates for non-athletes are imprecise. These group differences should therefore be interpreted as preliminary and hypothesis-generating only. Future studies with larger, balanced cohorts are needed to confirm or refute these patterns. Second, we utilized a markerless motion capture system. While validated for sagittal plane movements, coronal plane measures (e.g., knee valgus) may have lower precision than marker-based systems. We mitigated this by focusing on correlations with LESS rather than absolute clinical diagnostic values. Third, the absence of force plates meant that landing phases were defined by kinematic proxies. Although visually verified, this may introduce minor temporal inaccuracies compared to kinetic thresholds.

## 5. Conclusions

In healthy young men, an integrated LESS–markerless 3D–EMG approach revealed that (i) LESS is independently associated with left-side dynamic knee valgus; (ii) bilateral hamstring acceleration-phase activity negatively predicts right-side valgus, suggesting a potential reactive compensatory mechanism; and (iii) exploratory comparisons indicate that soccer/taekwondo athletes may employ posterior-chain-dominant strategies (lower RF deceleration and lower RF/BF ratios) and exhibit smaller left-side valgus than non-athletes. These findings underscore the potential importance of hamstring preactivation for knee alignment during the landing-to-re-acceleration transition. Overall, this study supports the utility of combining field screening with markerless 3D and EMG to refine risk profiling, though the results regarding group differences should be interpreted as preliminary due to the unequal sample size. Future longitudinal and intervention studies employing high-speed synchronized EMG–kinematics–force measurements are needed to confirm these mechanisms and optimize training prescriptions.

## Figures and Tables

**Table 1:** Analysis of Variance According to Sport Type.

Variable		Soccer ^a^	Taekwondo ^b^	General ^c^	f	η^2^	poc
LESS Score		4.22 ± 1.56	5.25 ± 1.67	4.67 ± 2.81	0.860	0.056	-
Right	Valgus	−10.48 ± 6.54	−12.23 ± 3.30	−7.20 ± 2.21	0.974	0.063	-
Right	RF Acc	495.12 ± 226.36	518.68 ± 207.14	461.27 ± 262.24	0.066	0.005	-
Right	BF Acc	356.68 ± 188.75	427.58 ± 261.86	214.23 ± 110.55	1.096	0.07	-
Right	RF Dec	140.16 ± 150.00	514.77 ± 401.69	266.80 ± 310.42	4.566 *	0.239	a < b
Right	BF Dec	262.36 ± 338.38	298.58 ± 326.73	67.70 ± 60.77	0.570	0.038	-
Right	RF/BF Acc Ratio	1.72 ± 1.33	1.67 ± 0.99	2.72 ± 1.77	0.782	0.051	
Right	RF/BF Dec Ratio	1.21 ± 1.10	4.47 ± 4.45	3.85 ± 2.03	4.240 *	0.006	a < c
Left	Valgus	−12.07 ± 2.44	−11.17 ± 3.89	−7.85 ± 2.28	3.705 *	0.203	a < c
Left	RF Acc	518.57 ± 194.97	391.27 ± 87.96	514.97 ± 169.03	1.207	0.077	-
Left	BF Acc	385.26 ± 210.07	488.30 ± 136.12	177.37 ± 185.96	2.629	0.153	b > c
Left	RF Dec	114.43 ± 71.08	54.45 ± 18.25	271.83 ± 221.15	5.774 **	0.285	a,b < c
Left	BF Dec	420.21 ± 420.89	512.55 ± 381.00	147.60 ± 213.82	0.869	0.057	-
Left	RF/BF Acc Ratio	2.38 ± 2.68	0.84 ± 0.25	8.92 ± 11.75	3.720 *	0.204	a,b < c
Left	RF/BF Dec Ratio	1.17 ± 1.33	0.17 ± 0.16	5.79 ± 7.46	5.006 *	0.257	a,b < c

Note: ^a^, Soccer; ^b^, Taekwondo; ^c^, Non-athlete (General). Letters in the ‘poc’ column indicate significant differences between groups (Bonferroni post hoc test). Abbreviations: RF, Rectus Femoris; BF, Biceps Femoris; Acc, acceleration; Dec, deceleration. * *p* < 0.05, ** *p* < 0.01.

**Table 2:** Correlation Coefficients with LESS Score.

Variable		r	*p*
**Right**	Valgus	−0.453	0.018 *
**Right**	RF Acc	−0.358	0.093
**Right**	BF Acc	0.355	0.097
**Right**	RF Dec	0.058	0.792
**Right**	BF Dec	0.159	0.470
**Right**	RF/BF Acc Ratio	−0.541	0.008 **
**Right**	RF/BF Dec Ratio	0.006	0.980
**Left**	Valgus	−0.662	0.000 ***
**Left**	RF Acc	−0.231	0.289
**Left**	BF Acc	0.180	0.412
**Left**	RF Dec	−0.073	0.739
**Left**	BF Dec	0.194	0.375
**Left**	RF/BF Acc Ratio	−0.302	0.162
**Left**	RF/BF Dec Ratio	−0.251	0.248

RF, Rectus Femoris; BF, Biceps Femoris; Acc, acceleration; Dec, deceleration. * *p* < 0.05, ** *p* < 0.01, *** *p* < 0.001.

**Table 3:** Multiple Regression Models Predicting LESS.

Variable	B (95% CI)	SE	β	t	*p*
Right Valgus	−0.072 (−0.167, 0.023)	0.046	−0.245	−1.564	0.131
Left Valgus	−0.308 (−0.481, −0.135)	0.084	−0.574	−3.668	0.001 ***

R^2^ = 0.491 (Adjust R^2^ = 0.448). *** *p* < 0.001.

**Table 4:** Multiple Regression Models Predicting Valgus.

Variable		B (95% CI)	SE	β	t	*p*
**Right**						
	RF Acc	−0.010 (−0.033, 0.012)	0.010	−0.366	−0.989	0.342
	BF Acc	−0.021 (−0.041, −0.001)	0.009	−0.698	−2.237	0.045 *
	RF Dec	0.010 (−0.003, 0.023)	0.006	0.486	1.714	0.112
	BF Dec	0.012 (−0.003, 0.027)	0.007	0.627	1.779	0.101
**Left**						
	RF Acc	0.014 (−0.013, 0.042)	0.012	0.408	1.158	0.269
	BF Acc	−0.020 (−0.039, 0.000)	0.009	−0.633	−2.211	0.047 *
	RF Dec	−0.010 (−0.038, 0.018)	0.013	−0.175	−0.753	0.466
	BF Dec	0.000 (−0.011, 0.012)	0.005	0.028	0.089	0.930

RF, Rectus Femoris; BF, Biceps Femoris; Acc, acceleration; Dec, deceleration. R^2^ = 0.650 (Adjust R^2^ = 0.387). * *p* < 0.05.

## Data Availability

Derived data supporting the findings of this study are available from the corresponding author on request.
